# Dr. Joseph Goffin Bailey

**Published:** 1910-06

**Authors:** 


Dr. Joseph Goffin Bailey, of Santa Ana, Cal., died at his home in that city April 29, 1910, of heart disease, aged 65 years. He graduated from the University of Buffalo, Medical Department, in 1871.

